# *Ankk1* Loss of Function Disrupts Dopaminergic Pathways in Zebrafish

**DOI:** 10.3389/fnins.2022.794653

**Published:** 2022-02-08

**Authors:** Adele Leggieri, Judit García-González, Jose V. Torres-Perez, William Havelange, Saeedeh Hosseinian, Aleksandra M. Mech, Marcus Keatinge, Elisabeth M. Busch-Nentwich, Caroline H. Brennan

**Affiliations:** ^1^School of Biological and Behavioural Sciences, Queen Mary University of London, London, United Kingdom; ^2^Department of Genetics and Genomic Sciences, Icahn School of Medicine at Mount Sinai, New York, NY, United States; ^3^Department of Brain Sciences, UK Dementia Research Institute, Imperial College London, London, United Kingdom; ^4^Centre for Discovery Brain Sciences, The University of Edinburgh, Edinburgh, United Kingdom; ^5^Department of Medicine, Cambridge Institute of Therapeutic Immunology and Infectious Disease, University of Cambridge, Cambridge, United Kingdom

**Keywords:** *ANKK1*, *DRD2*, dopaminergic system, addiction, amisulpride, apomorphine

## Abstract

Ankyrin repeat and kinase domain containing 1 (ANKK1) is a member of the receptor-interacting protein serine/threonine kinase family, known to be involved in cell proliferation, differentiation and activation of transcription factors. Genetic variation within the *ANKK1* locus is suggested to play a role in vulnerability to addictions. However, *ANKK1* mechanism of action is still poorly understood. It has been suggested that *ANKK1* may affect the development and/or functioning of dopaminergic pathways. To test this hypothesis, we generated a CRISPR-Cas9 loss of function *ankk1* zebrafish line causing a 27 bp insertion that disrupts the *ankk1* sequence introducing an early stop codon. We found that *ankk1* transcript levels were significantly lower in *ankk1* mutant (*ankk1^27*ins*^*) fish compared to their wild type (*ankk1*^+/+^) siblings. In *ankk1*^+/+^ adult zebrafish brain, ankk1 protein was detected in isocortex, hippocampus, basolateral amygdala, mesencephalon, and cerebellum, resembling the mammalian distribution pattern. In contrast, ankk1 protein was reduced in the brain of *ankk1^27*ins/*27*ins*^* fish. Quantitative polymerase chain reaction analysis revealed an increase in expression of *drd2b* mRNA in *ankk1^27*ins*^* at both larval and adult stages. In *ankk1*^+/+^ adult zebrafish brain, drd2 protein was detected in cerebral cortex, cerebellum, hippocampus, and caudate homolog regions, resembling the pattern in humans. In contrast, drd2 expression was reduced in cortical regions of *ankk1^27*ins/*27*ins*^* being predominantly found in the hindbrain. No differences in the number of cell bodies or axonal projections detected by anti-tyrosine hydroxylase immunostaining on 3 days post fertilization (dpf) larvae were found. Behavioral analysis revealed altered sensitivity to effects of both amisulpride and apomorphine on locomotion and startle habituation, consistent with a broad loss of both pre and post synaptic receptors. *Ankk1^27*ins*^* mutants showed reduced sensitivity to the effect of the selective dopamine receptor antagonist amisulpride on locomotor responses to acoustic startle and were differentially sensitive to the effects of the non-selective dopamine agonist apomorphine on both locomotion and habituation. Taken together, our findings strengthen the hypothesis of a functional relationship between *ANKK1* and *DRD2*, supporting a role for *ANKK1* in the maintenance and/or functioning of dopaminergic pathways. Further work is needed to disentangle *ANKK1*’s role at different developmental stages.

## Introduction

Addiction or substance use disorder (SUD) is a complex condition characterized by the uncontrolled use of drugs despite harmful and adverse consequences. Although environmental factors such as early life trauma, altered family structure, social pressure, and isolation during childhood increase the risk of developing SUDs (Wong et al., 2013; Baarendse et al., 2014; Koeneke et al., 2020), genetic factors also contribute to the liability of the disorder, with heritability estimates ranging from 40 to 60% (see Prom-Wormley et al., 2017 and Lopez-Leon et al., 2021, for reviews).

The Taq1A polymorphism (rs1800497) is one of the most extensively studied genetic variants in relation to drug addiction (Blum et al., 1990; Noble, 2003; Ponce et al., 2009; Wang et al., 2013) and psychiatric disorders (Neville et al., 2004; Hoenicka et al., 2010). Taq1A is located within exon 8 of the ankyrin repeat and kinase domain containing 1 (*ANKK1*) gene, causing a single nucleotide C(A2)/T(A1) change (Neville et al., 2004) resulting in a glutamate to lysine substitution (Glu713Lys) in the C-terminal ankyrin repeat domain, which might lead to a change in protein function (Völter et al., 2012).

Ankyrin repeat and kinase domain containing 1 is a serine/threonine kinase belonging to the receptor-interacting protein (RIP) family. RIP kinases are important regulators of cell survival, death, and differentiation (Meylan and Tschopp, 2005; Declercq et al., 2009; Zhang et al., 2010; Humphries et al., 2015). *ANKK1* maps to chromosome 11q22-q23 (chr11: 11,338,038–113,400,418; GRCh38/hg38) in a 512 kb gene cluster that includes the neural cell adhesion molecule 1 (*NCAM1*), tetratricopeptide repeat domain 12 (*TTC12*) and dopamine receptor 2 (*DRD2*) genes (Neville et al., 2004; Mota et al., 2012). The *NCAM-TTC12-ANKK1-DRD2* (NTAD) cluster is conserved among the vertebrates and has been proposed to be involved in neurogenesis and in the development of dopaminergic pathways (Yi et al., 2007; España-Serrano et al., 2017; Rubio-Solsona et al., 2018; Koeneke et al., 2020). In the adult mouse brain, ANKK1 protein is expressed in neural stem cells, in post-mitotic neurons and in migrating neuroblasts (España-Serrano et al., 2017; Koeneke et al., 2020), hinting at a role in neuronal differentiation and migration.

Ankyrin repeat and kinase domain containing 1 may be particularly important for the correct development and regulation of dopaminergic pathways, since DRD2 protein expression, density and binding is reduced in the striatum of Taq1A A1 allele carriers (Noble, 2003; Neville et al., 2004; Klein et al., 2007; Savitz et al., 2013). It has been suggested that *ANKK1* variants may influence addiction vulnerability by affecting differentiation, migration, and/or connectivity of dopaminergic neurons during development, and by modulating dopaminergic function in the brain during adulthood.

To test the hypothesis that *ANKK1* modulates development and function of the dopaminergic system, we generated a CRISPR-Cas9 loss of function line for *ANKK1* (referred to as *ankk1^27*ins*^*) using the zebrafish as a model organism. Zebrafish is an established model for developmental genetic studies (see Link and Megason, 2008 and Sakai et al., 2018, for reviews) and show conservation of pathways associated with responses to drugs of abuse (Mathur and Guo, 2010; Clark et al., 2011; Klee et al., 2011, 2012; Stewart et al., 2011; Parker et al., 2013; Bradford et al., 2017). We examined ankk1 and drd2 protein expression in the brains of wild type (*ankk1*^+/+^) and mutant (*ankk1^27*ins*^*) adult fish using immunohistochemistry and quantitative real time polymerase chain reaction (qPCR). We show that ankk1 and drd2 proteins are expressed in similar domains in the zebrafish brain as in human. Ankk1 transcript and protein levels were reduced in the brains of *ankk1* mutants. Drd2 protein levels were also reduced. In contrast, *drd2b* transcript levels were found to be increased at both larval and adult stages. We observed no differences (*ankk1*^+/+^ versus *ankk1^27*ins/*27*ins*^*) in the number of cell bodies nor axonal projections when performing anti-tyrosine hydroxylase immunostaining on 3 dpf zebrafish larvae. Behavioral analysis revealed an effect of genotype on baseline locomotion but not on anxiety-like responses. *Ankk1^27*ins*^* mutants showed reduced sensitivity to the effect of the selective dopamine receptor antagonist amisulpride on locomotor responses to acoustic startle and were differentially sensitive to the effects of the non-selective dopamine agonist apomorphine on both locomotion and habituation.

## Materials and Methods

### Animal Maintenance

Wild type Tübingen (TU) strain zebrafish were housed in a recirculating system (Tecniplast, United Kingdom) on a 14 h:10 h light/dark cycle and a constant temperature of 28°C. Fish were fed twice daily with ZM-400 fry food (Zebrafish Management Ltd., Winchester, United Kingdom) in the morning, and brine shrimp in the afternoon.

Breeding was set up in the evening, either in sloping breeding tanks (Tecniplast, United Kingdom) or in tanks equipped with a container with marbles to isolate eggs from progenitors. For experiments where the developmental stage of larvae was important, we placed barriers between the fish to keep them isolated in the breeding tank. The following morning, barriers were removed to allow spawning.

Eggs were collected in Petri dishes the following morning, sorted fertile from infertile, and then incubated at 28°C (max 50 eggs/dish). Dishes were checked daily to ensure consistent developmental stage across groups. If reared, larvae were moved to the recirculating system at 5 dpf and fed as stated above.

All procedures were carried out under license in accordance with the Animals (Scientific Procedures) Act, 1986 and under guidance from the Local Animal Welfare and Ethical Review Board at Queen Mary University of London.

### Generation of *Ankk1* Loss of Function Zebrafish Line

Selection of target site and design of guide RNAs (crRNA) was as described previously (Keatinge et al., 2021). The crRNA was designed to target a *Bst*NI restriction enzyme site [**CCC**TGGATAATCTCCTTAGCAAT (PAM sequence in bold, restriction site underlined)]. 1 nL of a solution containing 62.5 ng/μl crRNA (Sigma-Aldrich/Merck, Darmstadt, Germany), 62.5 ng/μl tracrRNA (TRACRRNA05N, Sigma-Aldrich/Merck, Darmstadt, Germany), and 5 μM Cas9 (NEB M0386M, NEB Ltd., United Kingdom), was injected in one-cell stage zebrafish embryos (wild-type, TU). Around 100 embryos were injected and approximately 50 were raised to identify founders.

Founder carriers were identified by polymerase chain reaction (PCR) from genomic DNA (ankk1_Forward, 5′ – TCCAAAATT GGAAGAATGAAGTT – 3′; ankk1_Reverse, 5′ – GCAGAAA GTTCATACCCATCG – 3′). Pairs of fish carrying the same mutation were identified and reared over three generations. F_3_ heterozygous carriers were then in-crossed to obtain F_4_ fish for characterization.

Quantitative real time polymerase chain reaction and immunohistochemistry were used to confirm reduction of *ankk1* mRNA and protein expression.

### RNA Isolation and cDNA Synthesis

Five pools of 16 zebrafish larvae combined according to their genotype (wild type and heterozygous), four pools of 16 zebrafish larvae (homozygous), and 6 whole adult brain (males) for each genotype were collected in RNase free 1.5 mL Eppendorf tubes, water was removed, and samples snap frozen (−80°C) until usage. Total RNA was isolated using TRIzol reagent (Thermo Fisher Scientific, United States) following manufacturer’s instructions. Briefly, after homogenization, RNA was isolated by precipitation, rinsed, and resuspended in RNase free water. Total RNA was then quantified using BioDrop (Biochrom Ltd., United Kingdom), and up to 1 μg was reverse transcribed to cDNA using the ProtoScript II First Strand cDNA Synthesis Kit (NEB Ltd., United Kingdom) following manufacturer’s protocol. Resulting cDNA yield and quality were also evaluated using BioDrop (Biochrom Ltd., United Kingdom).

### Quantitative Real-Time PCR

Quantitative real time polymerase chain reaction was performed using the Power SYBR Green PCR Master Mix (Applied Biosystems, Thermo Fisher Scientific, United Kingdom) and in a Bio-Rad 96-well qPCR machine (CFX96 Touch Real-Time PCR Detection System). All reactions were carried out in triplicate. Actin – β 2 (*actb2*), ribosomal protein L13a (*rpl13a*), and eukaryotic translation elongation factor 1 alpha 1 – like 1 (*efl*), were employed as reference genes. Amplification conditions were as follows: 95°C × 5 min, 50 cycles of 95°C × 10 s, 60°C × 12 s, and 72°C × 12 s. A*nkk1* primers were designed downstream of the CRISPR insertion to detect disruption in *ANKK1* mRNA levels as a consequence of the mutation. The qPCR product spans the genomic region chr15: 22,084,486–22,085,215, whereas the CRISPR insertion is located in chr15: 22,078,972 (GRCz10/danRer10 Assembly). Accession numbers, primer sequences and amplification efficiencies for all the reference and target genes can be found in Supplementary Table 1.

### Immunohistochemistry on Adult Brain Sections

As *ankk1* transcript level was similarly reduced in both heterozygous and homozygous mutants we compared protein distribution in wild types and homozygous mutants only. Immunohistochemistry was conducted on paraffin embedded zebrafish brains from male wild types and homozygous *ankk1^27*ins/*27*ins*^*. Fish were culled by overdose of tricaine prior to head removal. Brains were dissected and fixed in 4% paraformaldehyde (PFA, Sigma, Gillingham, United Kingdom) in 1x phosphate buffered saline (PBS), overnight (ON) at 4°C. Brains were then rinsed in 1x PBS and dehydrated in ascending ethanol series (15 min in each of 30, 50, 70, 80, 90, and 100% ethanol) and embedded in paraffin. Transverse sections of 12 μm thickness were cut using a microtome (Leica, Wetzlar, Germany). To perform immunohistochemistry, slides were de-waxed in xylene (twice, 10 min each), rehydrated in descending ethanol series (2 × 5 min in absolute ethanol, then 90, 80, and 70%, 5 min each), and rinsed in distilled water for 5 min. An antigen retrieval step was performed with citrate buffer solution (0.01 M, pH 6.00): citrate buffer was pre-heated (95–100°C), slides were immersed in the solution, covered with a lid (loosely) and incubated for 30 min. Sections were cooled at room temperature (RT) for 20 min, rinsed in 1x PBS for 5 min and endogenous peroxidase activity was quenched with 3% H_2_O_2_, 20 min at RT. Slides were washed three times in 1x PBS, 5 min each time, and incubated in blocking solution (BS) (10% normal goat serum, and 2 μg/μL bovine serum albumin in 1x PBS) for 30 min in a humid chamber at RT. Slides were subsequently incubated with anti-ankk1 Rabbit pAb (A16178, ABclonal), or anti-drd2 Rabbit pAb (A12930, ABclonal), 1:200 in BS, ON at 4°C. The day after, sections were well washed (5 × 10 min) in 1x PBS, and incubated with ImmPRESS ^®^ HRP Goat Anti-Rabbit IgG Polymer Detection Kit (MP-7451-15, Vector), at RT. The immunoreactivity of the cells was visualized using freshly prepared solutions of 3,3’-diaminobenzidine tetrahydrochloride (0.05% in 1x PBS) activated with a solution of 0.03% H_2_O_2_. When the desired staining was obtained, sections were well washed in 1x PBS (3 × 10 min), then 3 × 10 min in distilled water, dehydrated in ascending ethanol series (70, 80, 90, and 100%, 5 min each), cleared in xylene (twice, 5 min each) and mounted with mounting medium.

### Immunohistochemistry on Whole Mount Zebrafish Larvae

As *ankk1* transcript level was similarly reduced in both heterozygous and homozygous mutants we again compared protein distribution in wild type and homozygous mutants only. Fluorescent immunohistochemistry was carried out in 3 dpf larvae from *ankk1*^+/+^, and *ankk1*^27ins/27ins^ in-crosses. To prevent skin pigmentation, embryos were incubated in 0.2 mM of 1-phenyl 2-thiourea (PTU) (Sigma, Gillingham, United Kingdom) from 24 h after fertilization. When they reached the desired age (3 dpf), larvae were fixed in 4% PFA (Sigma, Gillingham, United Kingdom) to avoid tissue degradation ON at 4°C. The following day, larvae were rinsed in PBT (1xPBS, 0.05% Tween 20 v/v) supplemented with dimethyl sulfoxide (DMSO, 1% v/v) and Triton X-100 (0.1% v/v), three times 5 min each. After washes, larvae were permeabilized using proteinase K (0.02 μg/μL), for 30 min at 37°C. Larvae were then rinsed again in PBT/DMSO/Triton X-100, three times 5 min each, and incubated in BS supplemented with Tween-20 (0.05% v/v) and sodium azide (0.02% w/v) ON at 4°C. Larvae were subsequently incubated with rabbit polyclonal anti-tyrosine hydroxylase (anti-TH) primary antibody (1:200; Sigma, Gillingham, AB152). The primary antiserum was detected with anti-rabbit Alexa 546-conjugated secondary antibody (1:250, A11010, Thermo Fisher Scientific, Loughborough, United Kingdom). Larvae were cleared in 80% glycerol in 1x PBS and mounted with low melting point agarose in a sandwich of one large coverslip (24 × 60 mm), with medium size coverslips (22 × 22 mm) used as spacers.

### Imaging Acquisition and Processing

For light immunohistochemistry, pictures were acquired by Leica DMRA2 upright epifluorescent microscope with color QIClick camera (Leica, Wetzlar, Germany) and processed with Velocity 6.3.1 software (Quorum Technologies Inc).

Immunofluorescence pictures were acquired with Leica SP5 confocal microscope (Leica, Wetzlar, Germany). Confocal Z stacks were recorded under the same conditions (speed 400 Hz, resolution 2048 × 2048, line average 1 and accumulation 1, frame average 6 and accumulation 1) using diode laser. Areas of interest for quantification were isolated, making sure that for all the individuals the same number of Z stacks (covering the same dorsal/ventral distance) were included. The number of cells and axon peaks were calculated using ImageJ (National Institutes of Health, United States). To calculate the intensity of the axon peak, a line was drawn from the medulla oblongata interfascicular zone and vagal area to the locus coeruleus. Then the average intensity along the midline was calculated.

Anatomical structures were identified according to the Neuroanatomy of the Zebrafish Brain by Wullimann et al. (1996).

### Behavioral Assays

#### Forced Light/Dark Test

Patterns of locomotor activity of 5 dpf mutant and wild type zebrafish larvae were investigated in a forced light dark assay as described previously (Glazer et al., 2018). After an initial 10 min period of dark (baseline), larvae were exposed to two light/dark cycles of 10 min each. Tests were conducted between 9 a.m. and 4 p.m. Larvae were placed in individual wells of a 48-multiwell plate in a volume of 300 μL. To reduce stress due to manipulation, fish were acclimatized for at least 1 h in ambient light before testing. Distances traveled were recorded using EthoVision XT software (Noldus Information Technology, Wageningen, Netherlands). Data were exported in both 1 min and 1 s time bins and analyzed with R programming language (R Core Team, 2020), as previously described (García-González et al., 2021).

#### Response and Habituation to Acoustic Startle

We assessed the response and habituation to acoustic startle stimuli in wild type and mutant larvae at 5 dpf in the presence and absence of the dopamine D2/D3 receptor antagonist amisulpride and non-selective dopamine receptor agonist apomorphine. Behavioral assays were conducted between 9 a.m. and 4 p.m. Larvae were placed in individual wells of a 48 multi-well plate. A control (0.05% DMSO) or treatment (0.01 mg/L amisulpride or 0.2 mg/L apomorphine in 0.05% DMSO) dose was added to each well in a final volume of 300 μL. Larvae were acclimatized for 1 h before testing. Then, plates were placed in a DanioVision Observation Chamber containing a dedicated tapping device (Noldus Information Technology, Wageningen, Netherlands). After 5 min of acclimation, larvae were subjected to 20 sound/vibration stimuli over 40 s (2 s intervals between each stimulus). For all experiments, distance traveled was recorded using EthoVision XT software (Noldus Information Technology, Wageningen, Netherlands), and data were outputted in 1 s time bins and analyzed as previously described (García-González et al., 2021).

### Statistical Analysis

For qPCR, relative mRNA expressions were calculated using a modified version of the Pfaffl method (Pfaffl, 2001) to account for multiple reference genes and slight variation in primer amplification efficiency (Hellemans et al., 2007; Evans et al., 2021). Differences in gene expression were assessed using a one-way ANOVA with genotype as independent variables. Data were log 10 transformed to achieve normal distribution for parametric statistical analysis. Log 10 transformed descriptive statistics are presented in Supplementary Table 2. We used the R package “*emmeans”* (R programming language, R Core Team, 2020) to calculate appropriate means and 95% confidence intervals for the contrasts within each gene of interest with a Dunn-Sidak adjustment (Šidák, 1967). *P*-values were generated and adjusted for multiple comparison using the “*pairs”* function (Supplementary Table 3).

For behavioral analysis, all data were analyzed with R programming language (R Core Team, 2020). For models where distance moved was a response variable, we fitted data to mixed linear models using the “*lme4*” package, and where proportion of responders was our response variable, we fitted data to beta regression models using the “*betareg*” package. In all instances, we used genotype (stimulus number, and drug treatment for acoustic startle assay) as fixed effects, and where permissible we used fish ID and day as random effects. As in García-González et al. (2021), we reported significant fixed effects as Type II Wald χ^2^ from models using the package “*car*,” *post hoc* Tukey’s tests were also conducted as necessary with the package “*emmeans*”.

## Results

**FIGURE 1:**
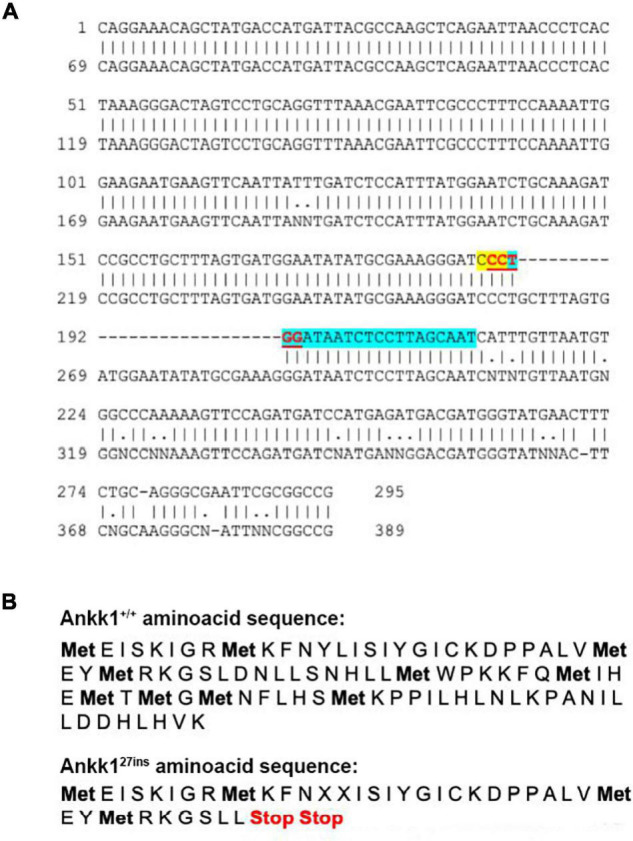
CRISPR-Cas9 generated a 27 bp insertion that disrupts the *ankk1* sequence **(A)** Comparison of wild type (top) and mutant (bottom) *ankk1* sequences. crRNA is highlighted in blue. PAM sequence is highlighted in yellow. The restriction site (that is disrupted in the F_0_ screening) appears in red, underlined. **(B)** Comparison of wild type (top) and mutant (bottom) amino acid sequences. Mutant sequence generates an early stop codon.

CRISPR-Cas9 generated a 27 bp insertion that disrupts the *ankk1* sequence introducing an early stop codon. Details are shown in Figure 1.

### Ankk1 Is Reduced at Both mRNA and Protein Levels in *Ankk1^27*ins*^* Fish

To confirm disruption of *ankk1*, we examined its expression at both mRNA and protein levels.

**FIGURE 2:**
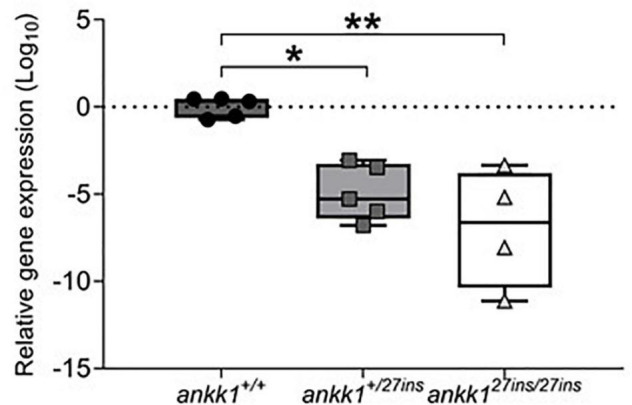
Depletion of mRNA transcripts in 5 days post fertilization zebrafish larvae. *ankk1*^+/+^ (dark gray), *ankk1^+/27*ins*^* (gray), and *ankk1^27*ins/*27*ins*^* (white). Data shows box and whiskers (5–95 percentile) and single samples (dots, squares, or triangles). Horizontal lines indicate group mean. Legend: *p ≤ 0.05, **p ≤ 0.01.

Transcript levels were significantly lower in *ankk1^27*ins*^* mutant fish compared to *ankk1*^+/+^ [*F*(2,11) = 13.86, *p* = 0.0010] (Figure 2). Within-family comparison showed that *ankk1* expression is significantly downregulated in both *ankk1^+/27*ins*^* (*p* = 0.0031) and *ankk1^27*ins/*27*ins*^* (*p* = 0.0004) larvae.

**FIGURE 3:**
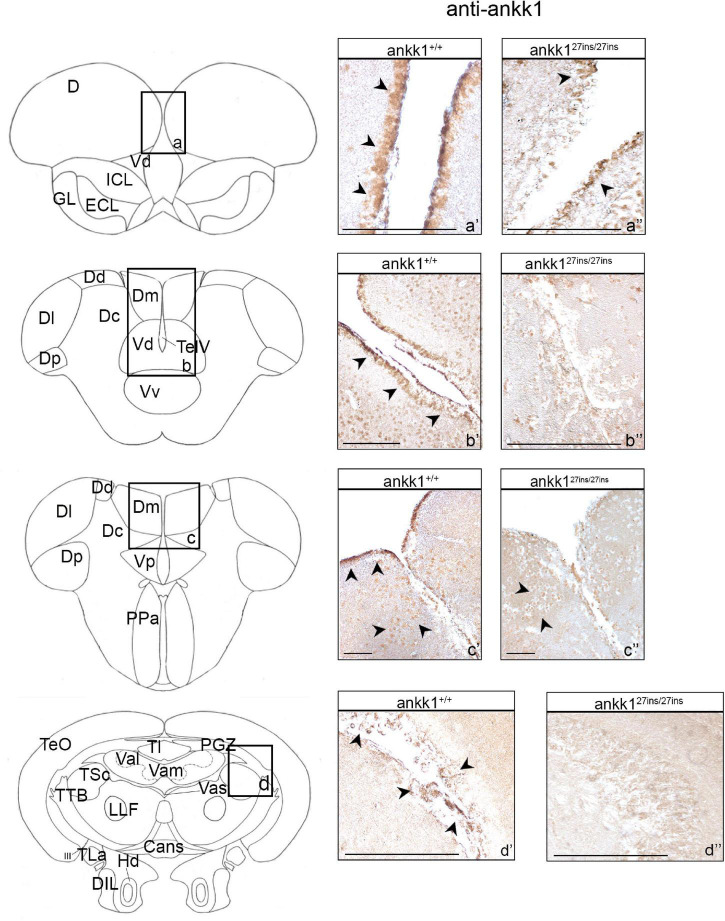
Ankk1 immunohistochemistry in adult zebrafish brain. On the right, ankk1 protein distribution in transverse sections of adult zebrafish brain, *ankk1*^+/+^ and *ankk1^27*ins/*27*ins*^*. On the left, schematic depiction of zebrafish brain, transverse section [adapted from Wullimann et al. (1996)]. **(a–d)** Boxes on the schematic depictions represent the region of the brain showed by the corresponding immunohistochemistry on the right; **(a’–c”)** forebrain ankk1 staining; **(d’,d”)**; midbrain ankk1 staining. Scale bars: **(c’,c”)**, 50 mm; **(b’)**, 100 mm; **(a’,a”,b”,d’,d”)**, 200 mm. Arrows indicate anti-ankk1 positive cells.

Ankk1 immunoreactivity in the brain of *ankk1^27*ins/*27*ins*^* versus *ankk1*^+/+^ was reduced at adult stage (Figure 3 and Supplementary Figure 1). In *ankk1*^+/+^ fish, numerous ankk1 immunoreactive cells were diffusely spread throughout the area dorsalis telencephalic (D) and in the area dorsalis lining the telencephalic ventricle (TelV), in the dorsal nucleus of the TelV (Vd) (Figures 3a’,b’ and Supplementary Figures 1a–c). Through most of the rostrocaudal extent of the area dorsalis, numerous clustered ankk1 immunoreactive cells were still observed in the areas of the Vd and of the medial zone of the dorsal telencephalic area (Dm) lining the TelV. In contrast, numerous scattered immunoreactive cells were detected centrally in the central zone (Dc) and in the Vd (Figure 3b’ and Supplementary Figure 1d). More caudally, in the area ventralis of telencephalon, few ankk1 immunoreactive cells were observed in the post-commissural nucleus (Vp) and in the Dm (Figure 3c’ and Supplementary Figures 1e,e’).

In *ankk1*^+/+^ fish midbrain, few immunoreactive cells were observed in the periventricular gray zone of the optic tectum (PGZ), in the vascular lacuna of area postrema (Vas), and in the lateral longitudinal fascicle (LLF) (Figure 3d’ and Supplementary Figures 1g–i).

In *ankk1*^+/+^ fish hindbrain, ankk1 immunoreactivity was detected dorsal to the inner arcuate fibers of the secondary octaval population (SO), in the intermediate reticular formation (IMRF), in the inner arcuate fibers (IAF), in the descending trigeminal root (DV), in the magnocellular octaval nucleus (MaON), and in the sensory root of the facial nerve (VIIs). Furthermore, numerous ankk1 immunoreactive cells were detected along the ventral lining of the hindbrain ventricle, in the longitudinally oriented nucleus of the griseum centrale (GC) (Supplementary Figures 1j–l).

When comparing ankk1 immunoreactivity of adult *ankk1*^+/+^ versus *ankk1^27*ins/*27*ins*^* brains, the staining was reduced in the periventricular forebrain regions (Figures 3a’–c’,a”–c”) and absent in the PGZ of the midbrain (Figures 3d’,d”) and the hindbrain fibers (Supplementary Figure 4A).

### *Ankk1* Loss of Function Alters Drd2 Protein Expression Levels in Adult Zebrafish Brain

As *ankk1* is proposed to regulate drd2 expression levels, we examined the expression pattern of drd2 protein in *ankk1*^+/+^ adult zebrafish brain and compared it with the expression pattern of *ankk1^27*ins/*27*ins*^*.

**FIGURE 4:**
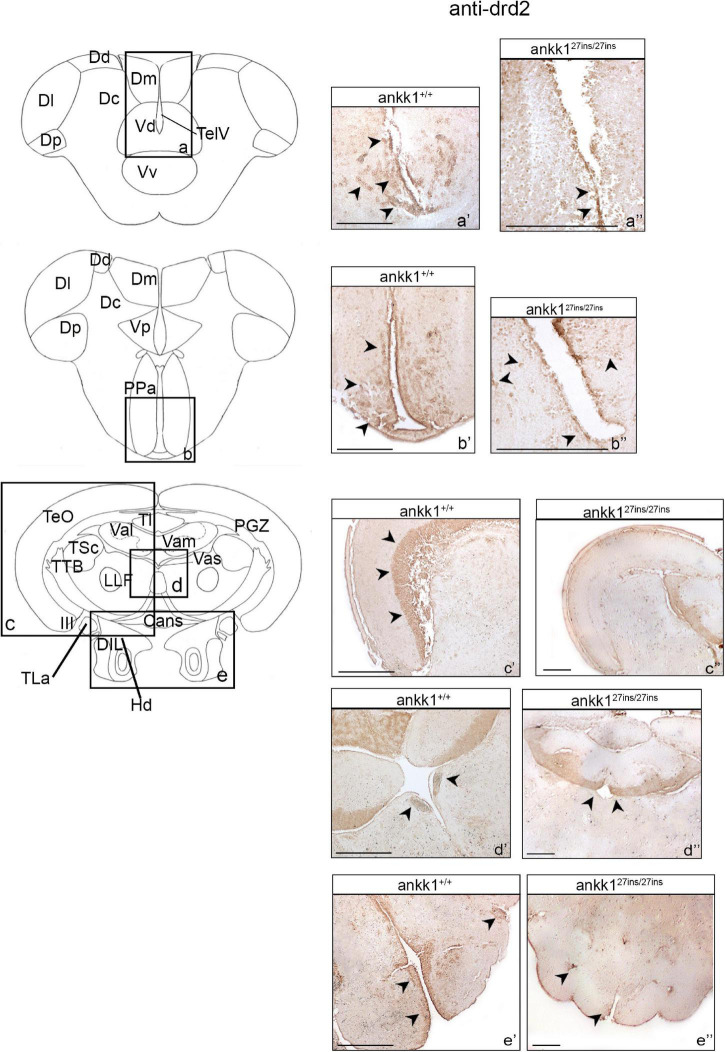
Drd2 immunohistochemistry in adult zebrafish brain. On the right, drd2 protein distribution in transverse sections of zebrafish brain, *ankk1*^+/+^ and *ankk1^27*ins/*27*ins*^*. On the left, schematic depiction of zebrafish brain, transversal section [adapted from Wullimann et al. (1996)]. **(a–e)** Boxes on the schematic depictions represent the region of the brain showed by the corresponding immunohistochemistry on the right; **(a’–b”)** forebrain drd2 staining; **(c’–e”)** midbrain drd2 staining. Scale bars: **(c”,d”,e’)**, 50 mm; **(a’–e’)**, 100 mm; **(b”)**, 200 mm. Arrows indicate anti-drd2 positive cells.

In *ankk1*^+/+^ fish forebrain, numerous drd2 immunoreactive cells clustered in the central area of D (Figure 4a’ and Supplementary Figure 2a). More caudally, drd2 immunoreactive cells were widely diffused in the Vd and ventral nucleus of the ventral telencephalic area (Vv) lining the TelV (Figure 4b’ and Supplementary Figures 2b,c). A few positive cells were also detected through most of the rostrocaudal extent of the area dorsalis, in the lateral and posterior zones of the dorsal telencephalic areas (respectively Dl and Dp) (Supplementary Figure 2b). Clustered drd2 immunoreactive cells were localized in the central nucleus of the ventral telencephalic area (Vc) and in the lateral olfactory tract (LOT) (Supplementary Figures 2b,c). Furthermore, a high density of drd2 immunoreactive cells was observed in the most ventral region of the Vv (Supplementary Figure 2c). In the periventricular area, numerous cells were found densely packed rostral to the anterior commissure in the Vd (Supplementary Figure 2d). In the diencephalic area, drd2 immunoreactivity was detected in specific areas: ventrally in the preoptic area, specifically in the anterior parvocellular preoptic nucleus (PPa), in the nucleus taeniae (NT) and in the ventral part of the entopeduncular nucleus (Supplementary Figures 2e,f).

In *ankk1*^+/+^ fish midbrain, drd2 immunoreactive cells were observed in the entire layer of the PGZ (Supplementary Figure 2g), in the axons perikarya in the layer of torus longitudinalis (Tl) (Supplementary Figures 2h,j), in the periventricular region of the lateral and medial divisions of valvula cerebelli (respectively Val and Vas) (Supplementary Figures 2h,j), and in the Vas (Supplementary Figure 2h). A thick layer of drd2 immunoreactive cells was observed in the dorsal zone of the periventricular hypothalamus (Hd) (Supplementary Figure 2i), whereas few positive cells were detected in the diffuse nucleus of the inferior lobe (DIL) (Supplementary Figure 2i).

In *ankk1*^+/+^ fish hindbrain, drd2 immunoreactive cells were detected in the entire area of the corpus cerebelli (CCe) (Supplementary Figure 2k).

We observed that drd2 immunoreactivity of adult *ankk1^27*ins/*27*ins*^* versus *ankk1*^+/+^ was drastically reduced. In *ankk1^27*ins/*27*ins*^* caudal forebrain region, drd2 immunoreactivity was detected only in a thin line of cells lining the TelV in the Vv (Figures 4a’,a”). In the diencephalic region, a clear reduction in drd2 protein expression was observed in the PPa and in the Vp (Figures 4b’,b”). In *ankk1^27*ins/*27*ins*^* midbrain, drd2 immunoreactivity was still present in the periventricular Val and Vam regions (Figures 4c’,c”), but completely absent in the Vas, PGZ, and in the Hd (Figures 4e’,d”). No differences were observed in the hindbrain (Supplementary Figure 4B).

### *Ankk1* Loss of Function Disrupts Dopaminergic Pathways in Zebrafish Larvae

As *ankk1* has been suggested to play a role in the development of dopaminergic pathways and in the differentiation of dopaminergic neurons, we examined mRNA expression levels of key components of the dopaminergic pathway by qPCR, and tyrosine hydroxylase expression levels by immunohistochemistry.

**FIGURE 5:**
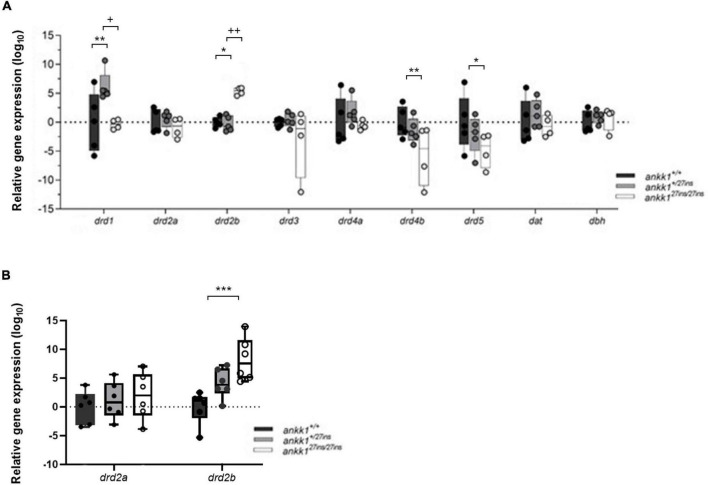
Quantification of dopaminergic gene expression. **(A)** Expression levels of *drd2a*, *drd2b*, *drd1*, *drd3*, *drd4a*, *drd4b*, *drd5*, *dat*, and *dbh* in 5 days post fertilization zebrafish larvae (*ankk1^+/+^, ankk1^+/27*ins*^, and ankk1^27*ins/*27*ins*^*) measured by qPCR. Each dot represents a pool of larvae (*n*_total_ = 80 larvae: 5 samples consisting of 16 larvae for *ankk1*^+/+^ and *ankk1^+/27*ins*^*, and 4 samples consisting of 16 larvae for each sample (ankk1^27*ins/*27*ins*^). **(B)** Expression levels of *drd2a* and *drd2b* in adult zebrafish whole brains. Each dot represents a single brain. Data are shown in box-whiskers plot (5–95 percentile). Legend: *p < 0.05 versus *ankk1*^+/+^; **p < 0.01 versus *ankk1*^+/+^; ***p < 0.0001 versus *ankk1*^+/+^; +*p* < 0.05 versus corresponding *ankk1^+/27*ins*^*; ++*p* < 0.01 versus corresponding *ankk1*^+/27*ins*^. Statistics for gene expression data and *P* value adjustment are provided in Supplementary Tables 2, 3.

First, we examined mRNA expression of the components of the dopaminergic pathway *drd2a, drd2b drd1, drd3, drd4a, drd4b, drd5, dat*, and *dbh* in 5 dpf zebrafish larvae (Figure 5). *P*-values, generated and adjusted for multiple comparison using the Dunn-Sidak method (Šidák, 1967), showed upregulation of *drd1* in *ankk1^+/27*ins*^* fish (*p* = 0.0016); upregulation of *drd2b* in *ankk1^27*ins/*27*ins*^* mutants, at both larval (*p* = 0.0128) and adult (*p* = 0.0004) stages; and downregulation of *drd4b* (*p* = 0.0083) and *drd5* (*p* = 0.0299) in *ankk1^27*ins/*27*ins*^* mutants.

**FIGURE 6:**
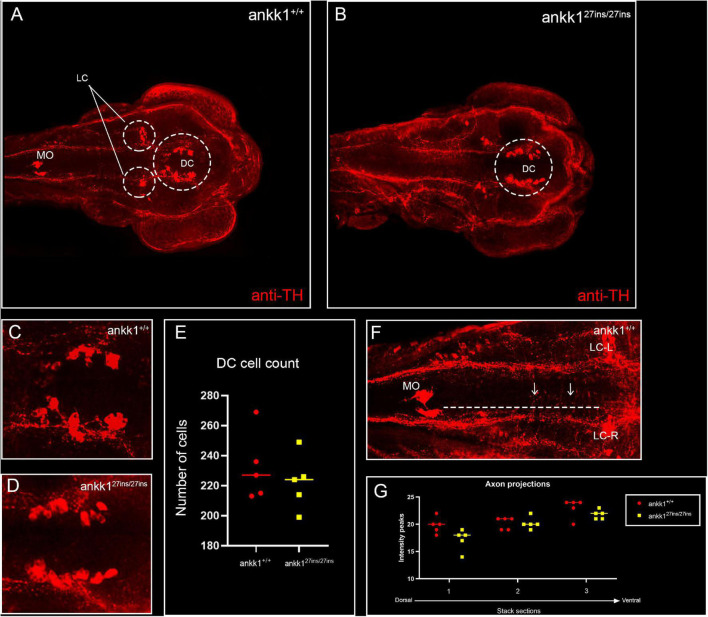
Anti-tyrosine hydroxylase (TH) immunolabeling on 3 days post fertilization zebrafish larvae. **(A,C)** *ankk1*^+/+^ and **(B,D)** *ankk1^27*ins*^*^/27*ins*^. **(A,B)** Maximum projection dorsal view of whole mount larvae. Circles indicate diencephalic dopaminergic cluster (DC), used for quantification of cell number **(E)**, and locus coeruleus (LC) used as landmark for determining the extent of the medial longitudinal catecholaminergic tract when quantifying the number of anti-TH labeled projections to the midline **(F,G)**. **(C,D)** Representative images of staining of DC used for cell quantification shown in **(E)**. **(F)** Example of sections used for quantification of dopaminergic projections shown in **(G)**. Projections were assessed from posterior to anterior using the LC and anterior extent of the medulla oblongata (MO) as landmarks [**(F)** dotted line, arrows indicate example of projections], and from dorsal to ventral [**(G)** stacks 1–3]. *N* = 5 samples × genotype group.

Next, to test the hypothesis whether *ankk1* possesses an important role in the formation of dopaminergic pathways during development more broadly, we performed fluorescence immunohistochemistry with tyrosine hydroxylase antiserum in 3 dpf zebrafish larvae and examined the number of cell bodies and axonal projections. We observed no significant differences in the number of cell bodies in the diencephalic dopaminergic cluster (*p* > 0.05) between *ankk1*^+/+^ and *ankk1^27*ins/*27*ins*^*, nor in the axon projections (*p* > 0.05) (Figure 6).

### *Ankk1^27*ins*^* Zebrafish Larvae Show Behavioral Differences Consistent With Altered Dopaminergic Signaling

**FIGURE 7:**
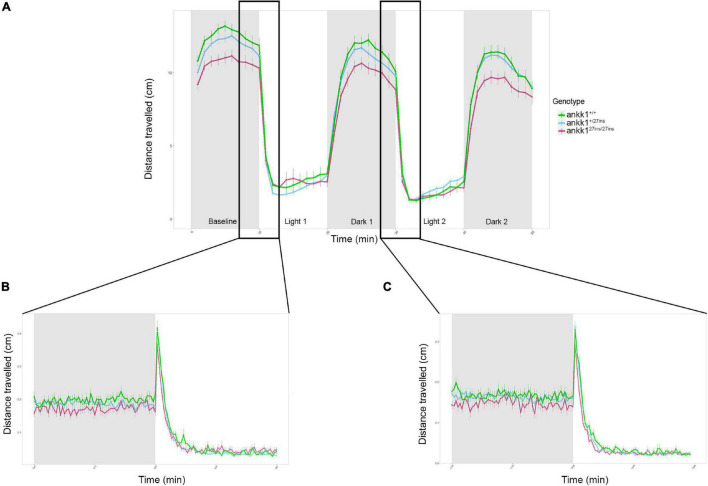
Forced light/dark test in 5 days post fertilization zebrafish larvae (*ankk1*^+/+^, *ankk1^+/27*ins*^*, *ankk1^27*ins/*27*ins*^*). **(A)** The assay consisted of 10 s of basal tracking, followed by two light/dark cycles of 10 min each. Dots represent mean distance traveled per minute. Error bars show standard error of the mean. **(B,C)** One-second time bins resolution plots of the dark/light transitions.

As the dopaminergic system plays a key role in stress response and regulation of anxiety levels (de la Mora et al., 2010), we assessed anxiety-like behavior in *ankk1*^+/+^ and *ankk1^27*ins*^* 5 dpf larvae, using a forced light dark transition assay. When the course of the test was examined as a whole (50 min), as well as when distances traveled were examined during light and dark periods separately, only time predicted distance traveled by zebrafish larvae (*p* < 0.0001). No significant differences were observed between genotypes (*p* > 0.05) (Figure 7A). However, when the baseline period (first 10 min of the experiment) was examined separately, distance traveled differed by time [Effect of time: χ^2^(1) = 25.14, *p* < 0.0001] and genotype [Effect of genotype: χ^2^(2) = 7.33, *p* = 0.025], such that *ankk1^27*ins/*27*ins*^* larvae moved significantly less than *ankk1*^+/+^ [(M*_*ankk*127*ins/*27*ins*_* = 1.09, SE = 0.0391), (M*_*ankk*1+/+_* = 1.23, SE = 0.0355) (*p* = 0.0188)].

On transitions from light to dark, all larvae sharply increased their locomotion and then steadily decreased it. On transition from dark to light, a rapid startle response resulting in a brief, sharp increase in locomotion was observed followed by a reduction in movement. The sharp increase in movement was only observable when examined at one second resolution (Figures 7B,C). We observed no significant differences between *ankk1* genotypes during light to dark, nor dark to light transitions (*p* > 0.05).

Another way of assessing the response to light changes is to evaluate the increase in locomotion during the light periods (measured as the slopes from min 10–20 for light period 1, and 30–40 for light period 2), and the decrease of locomotion during the dark periods (measured as the slopes from min 20–30 for dark period 1 and 40–50 for dark period 2). For both the two light and dark periods, despite apparent reduction in rate of recovery in mutant larvae (i.e., lower slopes), there were no significant differences between *ankk1* genotype groups (*p* > 0.05).

To test the impact of *ankk1* loss of function on dopamine regulated behavior associated with addiction vulnerability, we examined habituation to acoustic startle in 5 dpf larvae in the presence and absence of amisulpride and apomorphine. Habituation to acoustic startle is a measure of sensorimotor gating and is sensitive to modulation by dopaminergic agonists and antagonists (Quednow et al., 2006; Burgess and Granato, 2007; Halberstadt and Geyer, 2009; García-González et al., 2020).

**FIGURE 8:**
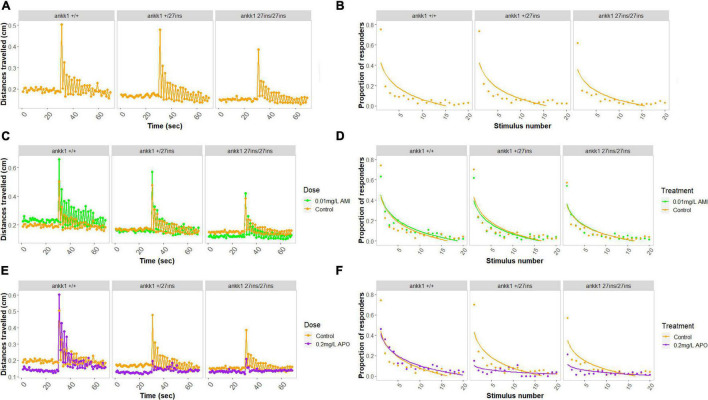
Habituation to acoustic startle response in 5 days post fertilization larvae (*ankk1^+/+^, ankk1^+/27ins^*, and *ankk1*^27ins/27ins^) in presence (0.01 mg/L) or absence (control) of amisulpride. The assay consisted of 60 s of basal tracking, followed by 20 acoustic stimuli within 2 s intervals. **(A,C,E)** Mean distances traveled by *ankk1^+/+^, ankk1^+/27ins^, ankk1^27ins/27ins^*. Sample size: *N* = 145 per genotype. **(B,D,F)** Proportion of individuals responding to each startle stimulus. *N* = 226 *ankk1*^+/+^_control_, *N* = *ankk1*^+/27ins^_control_, *N* = 226 *ankk1^27*ins*/27*ins*^*_control_, *N* = 226 *ankk1*^+/+^_AMI_, *N* = 145 *ankk1^+/27*ins*^*_AMI_, *N* = 145 *ankk1^27*ins/*27*ins*^*_AMI_, *N* = 145 *ankk1*^+/+^_APO_, *N* = 145 *ankk1^+/27*ins*^*_APO_, *N* = 145 *ankk1^27*ins/*27*ins*^*_APO_. Error bars show standard error of the mean.

In the absence of drugs, larvae showed a habituation response to repeated acoustic startle consistent with previous reports (García-González et al., 2020): 73% of wild type animals responded to the first acoustic stimulus, but only 4% responded to the last. We found a significant effect of genotype on distance traveled in the basal portion of the assay (Figure 8A) [Effect of genotype: χ^2^(2) = 15.8972, *p* < 0.001] whereby *ankk1^27*ins*^* fish moved significantly less than *ankk1*^+/+^ (Tukey’s pairwise comparisons: *ankk1*^+/+^-*ankk1^+/27*ins*^ p* = 0.05, *ankk1*^+/+^-*ankk1^27*ins/*27*ins*^ p* < 0.001).

During the stimulus events we observed a significant effect of stimulus number [Effect of stimulus number: χ^2^(19) = 2702.753, *p* < 0.0001], and a significant effect of genotype [Effect of genotype: χ^2^(2) = 19.380, *p* < 0.0001], on locomotion whereby *ankk1^27*ins/*27*ins*^* fish moved significantly less than *ankk1*^+/+^ (Tukey’s pairwise comparisons: *ankk1*^+/+^-*ankk1^27*ins/*27*ins*^ p* < 0.001). There was a genotype by stimulus number interaction [Effect of stimulus number: χ^2^(38) = 84.131, *p* < 0.0001] such that *ankk1^27*ins*^* habituated more slowly.

In the presence of amisulpride we saw no main effect of dose on basal locomotion. However, we detected a two-way interaction [Effect of genotype by dose interaction: χ^2^(2) = 13.5008, *p* < 0.01], such that amisulpride exposure increased the distance traveled in *ankk1*^+/+^ (Tukey’s pairwise comparison: *ankk1*^+/+^_0_._01mg/L_ – *ankk1*^+/+^_control_ *p* = 0.02), but not in *ankk1^+/27*ins*^* nor *ankk1^27*ins/*27*ins*^* genotypes.

During the stimuli (Figure 8C), we found an effect of dose on distance traveled [Effect of dose: χ^2^(1) = 40.689, *p* = 0.0001], and a genotype by dose interaction [Effect of genotype by dose interaction: χ^2^(2) = 9.036, *p* = 0.01], such that amisulpride exposure increased the distance traveled in *ankk1*^+/+^ (Tukey’s pairwise comparison: *ankk1*^+/+^_0_._01mg/L_ - *ankk1*^+/+^_control_ *p* = 0.02), but not in *ankk1^+/27*ins*^* nor *ankk1^27*ins/*27*ins*^* genotypes. We also found a marginal three-way interaction [Effect of genotype by dose by stimulus number interaction: χ^2^(38) = 51.632, *p* = 0.06], such that *ankk1^27*ins*^* habituate more slowly.

In the presence of apomorphine, we found a significant main effect of dose on basal locomotion [Effect of dose: χ^2^(1) = 18.6628, *p* < 0.0001], such that apomorphine treated fish moved less (Figure 8E).

During the stimuli (Figure 8E) we found a main effect of dose [Effect of dose: χ^2^(1) = 30.1613, *p* < 0.0001], where treated fish moved less, and a significant effect of stimulus number [Effect of stimulus number: χ^2^(19) = 2165.5652, *p* < 0.0001]. We detected a two-way interaction [Effect of genotype by dose: χ^2^(2) = 37.5713, *p* < 0.0001] and a three-way interaction [Effect of genotype by dose by stimulus number: χ^2^(38) = 68.5989, *p* < 0.01] (Figure 8B).

Since we discovered significant differences at the genotype and dose levels in basal locomotion, we calculated the proportion of responders to stimulus events using six discrete responder thresholds for each genotype by treatment group (Figures 8D,F). In the presence of amisulpride (Figure 8D), a lower proportion of *ankk1^27*ins/*27*ins*^* fish responded to stimulus events [Effect of genotype: χ^2^(2) = 5.1215, *p* < 0.05 (Tukey’s pairwise comparisons: *ankk1^+/27*ins*^* -*ankk1^27*ins/*27*ins*^ p* < 0.01, *ankk1*^+/+^ -*ankk1^27*ins/*27*ins*^ p* < 0.01)]. No main effect of dose or genotype by dose interaction was detected. In the presence of apomorphine (Figure 8F), a lower proportion of *ankk1^27*ins*^* fish responded to stimulus events [Effect of genotype: χ^2^(2) = 23.4687, *p* < 0.0001 (Tukey’s pairwise comparisons: *ankk1*^+/+^ -*ankk1^+/27*ins*^ p* < 0.0001, *ankk1*^+/+^ -*ankk1^27*ins/*27*ins*^ p* < 0.0001]. We observed a dose by stimulus number interaction [Effect of dose by stimulus number: χ^2^(1) = 7.3185, *p* < 0.01], and a genotype by dose interaction [Effect of genotype by dose: χ^2^(2) = 19.1113, *p* < 0.0001]; apomorphine reduced *ankk1^27ins^* response to acoustic startle.

## Discussion

In this study, we generated a CRISPR-Cas9 loss of function zebrafish line (*ankk1^27ins^*), to test the hypothesis that *ankk1* regulates the development and/or functioning of dopaminergic pathways. We confirmed that ankk1 protein is broadly expressed in regions of the zebrafish brain, and we showed reduction of *ankk1* mRNA and protein expression levels in *ankk1^27ins^* mutants. We found that *drd2b* mRNA was upregulated in *ankk1^27ins/27ins^* whole brain samples at larval and adult stages, but no differences in the number of dopaminergic neurons nor in axon pathfinding were detected in 3 dpf larvae. In contrast, drd2 protein expression was decreased in cortical regions, and was completely absent in specific midbrain areas of *ankk1^27ins/27ins^* adults. Finally, we reported that *ankk1^27ins/27ins^* larvae had reduced sensitivity to the dopaminergic D2/D3 antagonist amisulpride and were differentially sensitive to the effects of the non-selective dopaminergic agonist apomorphine. Taken together, our results support a role for *ankk1* in the maintenance and/or functioning of zebrafish dopaminergic pathways.

We confirmed *ankk1* loss of function by qPCR experiments. The numerical similarity in *ankk1* mRNA expression between *ankk1^+/27ins^* and *ankk1^27ins/27ins^* is intriguing. This could be due to (i) level of expression at the limit of resolution of our detection, or (ii) we may speculate that an autoregulatory mechanism of *ankk1* expression, such that reduction in active protein seen as a result of heterozygosity, leads to failure to maintain *ankk1* mRNA expression. However, differences in the expression of the components of the dopaminergic pathway between heterozygotes and homozygotes (e.g., *drd2b*) are not immediately consistent with this latter suggestion. Further experiments are required to address this hypothesis.

As we saw no significant differences between *ankk1^+/27ins^* and *ankk1^27ins/27ins^* in the levels of *ankk1* mRNA expression, we used *ankk1^27ins/27ins^* to assess differences in protein levels in wild type and mutant fish.

We describe the neuroanatomical distribution of ankk1 protein in the adult zebrafish brain. We detected ankk1 protein in many forebrain areas of the dorsal, medial, and ventral pallium, homologous to the mammalian isocortex, hippocampus and basolateral amygdala, respectively (Mueller, 2012; see Cheng et al., 2014 for review), and in the subpallium, homologous to the basal ganglia (see Cheng et al., 2014 for review). We also found ankk1 protein expression in the mesencephalon, and in the cerebellum. These results agree with findings in mice where Ankk1 protein is expressed in the prefrontal cortex, hippocampus, corpus callosum, thalamus, bulb, pons, mesencephalon, encephalic trunk, basal ganglia, cerebellum, and spinal cord (Hoenicka et al., 2010). In contrast, in *ankk1^27ins/27ins^* mutants, ankk1 protein expression was reduced in the forebrain and completely absent in the mid- and hindbrain. As the antibody recognizes an epitope before the stop-codon introduced by our mutation (A12930, ABclonal Immunogen Information), the low level of expression detected in the forebrain may reflect (i) incomplete destruction of the mRNA, (ii) non-specific binding or (iii) brain region specific alternative splicing. The use of different antibodies aiming to detect epitopes before and after the stop codon may be useful to interrogate the presence of region-specific splice variants.

In mammals, *ANKK1* has been proposed to modulate development and functioning of dopaminergic signaling pathways (Koeneke et al., 2020). Particularly, previous studies showed that the TaqA1 allele is associated with reduced DRD2 density in human striatum (Noble, 2003; Neville et al., 2004; Klein et al., 2007; Savitz et al., 2013). Dopaminergic receptors are well conserved among vertebrates. Due to the whole-genome duplication in teleosts (Glasauer and Neuhauss, 2014), zebrafish possess two *drd2* genes, *drd2a* (Chr15: 22,046,557–22,074,315) and *drd2b* (Chr5: 58,075,301–58,173,627), which show a sequence similarity of 71 and 66%, respectively, with human DRD2 (Boehmler et al., 2004). The antibody employed in our study recognizes an epitope which is common to both proteins. In agreement with previous findings (Noble, 2003; Neville et al., 2004; Klein et al., 2007; Savitz et al., 2013), adult *ankk1^27*ins/*27ins^* mutant fish showed reduced expression of drd2 protein in the pallium and subpallium. The zebrafish subpallium corresponds to the striatum of mammals, which has been shown to be involved in processes such as motor learning (Adrover et al., 2020). We also observed absence of drd2 protein in the hypothalamus of *ankk1^27ins/27ins^*. In zebrafish, hypothalamic dopaminergic neurons activate premotor circuits involved in swimming and sensorimotor integration (Barrios et al., 2020). These findings may explain the alterations in locomotor effects observed in our behavioral tests.

Drd2 protein antibody staining in adult mutant fish shows a different trend from drd2b mRNA expression at both larval and adult stages, where mutants had a higher *drd2b* gene expression. Such differences may be due to (i) biological processes (such as splicing, translational modifications/regulation, protein complex formation) that affect the relative quantities of mRNA and protein (Guo et al., 2008; Perl et al., 2017), (ii) loss of receptor function/signaling, which is often associated with compensatory increases in gene expression (Perdew et al., 2007), (iii) differences in the detection method.

Interestingly, *ANKK1* and *DRD2* form part of the NTAD genomic cluster in mammals (Yi et al., 2007; España-Serrano et al., 2017; Rubio-Solsona et al., 2018; Koeneke et al., 2020) but in zebrafish only *drd2a* (whose mRNA expression was not altered) maps to the NTAD region. It is therefore possible that in zebrafish, *drd2a* (but not *drd2b*) is part of the NTAD functional cluster associated with ANKK1 function, or that *drd2b* forms part of an interchromosomal NTAD gene cluster (Woods et al., 2005).

In addition to a role in regulation of *drd2* expression, *ankk1* has been proposed to play a role in neurogenesis and cell migration (Mota et al., 2012; España-Serrano et al., 2017; Koeneke et al., 2020). To test this hypothesis, we assessed the number of dopaminergic neurons in the diencephalic cluster and axonal projections from the medulla oblongata interfascicular zone and vagal area to the locus coeruleus of 3 dpf larvae but found no differences among *ankk1* genotypes. These findings argue against a role of *ankk1* in dopaminergic neurogenesis. Although our immunohistochemical analysis did not detect differences in axonal projections, our method would not have been sensitive enough to detect subtle changes (e.g., in dendrites and synapses) and it is possible that differences become more apparent at later stages of development.

We therefore conducted behavioral assays to analyze possible disruption of dopaminergic function in 5 dpf larvae using amisulpride and apomorphine, a selective D2/D3 dopamine receptor antagonist (Coukell et al., 1996) and a non-selective dopamine receptor agonist (Lees, 1993), respectively.

In mammals, dopamine receptors are coupled to G-proteins (see Beaulieu and Gainetdinov, 2011 for review). D1-like receptors (D1 and D5) have an excitatory effect on neurotransmission via activation of Gs proteins, whereas D2-like receptors (D2, D3, and D4) are coupled to Gi/o proteins mediating inhibitory neurotransmission (Beaulieu and Gainetdinov, 2011; Martel and Gatti McArthur, 2020). In rodents, low doses of D2/D3 receptor antagonists decrease locomotion via high affinity presynaptic receptors, whereas high doses increase locomotion via lower affinity postsynaptic receptors (Millan et al., 2004). Although binding affinities of D2/3 receptors in zebrafish are not established, and drugs could be metabolized by zebrafish in a different manner compared to mammals (Achenbach et al., 2018), a biphasic effect of D2/D3 receptor antagonists including amisulpride on locomotion has also been reported (Tran et al., 2015). The findings of Tran et al. (2015) confirm the involvement of both pre- and post-synaptic dopamine receptors in this species. Amisulpride has also been shown to increase habituation to acoustic startle in both humans (Quednow et al., 2006) and zebrafish (García-González et al., 2020).

Apomorphine is a short-acting non-selective dopamine receptor agonist that, similarly to amisulpride, binds to pre- and post-synaptic dopaminergic receptors in a dose-dependent manner (Auffret et al., 2018; Carbone et al., 2019). In mammals, apomorphine at low doses decreases locomotion via presynaptic receptors, whereas at high doses increases locomotion via postsynaptic receptors (Santos et al., 2018). Apomorphine treatment increases startle reactivity with no effect on the rate of habituation (Davis and Aghajanian, 1976; Geyer et al., 1978). In zebrafish it has been reported that apomorphine causes similar effects on locomotor activity as in mammals (Khalili et al., 2021, under review).

The altered sensitivity to effects of both amisulpride and apomorphine on locomotion and startle habituation seen here are consistent with a broad loss of drd2 as suggested by our immunohistochemistry. Amisulpride increased locomotion and increased habituation in *ankk1*^+/+^ but had no effect in *ankk1^27ins^* suggesting loss of post-synaptic receptors. Apomorphine decreased basal locomotion in both *ankk1*^+/+^ and, to a lesser extent, in *ankk1^27ins^* fish, increased locomotion in response to acoustic startle in *ankk1*^+/+^ fish, but decreased locomotion in response to acoustic startle in *ankk1^27ins^* fish. The decrease in basal locomotion in *ankk1^27ins^* mutants in the presence of apomorphine suggests action at presynaptic D2 and/or D3 autoreceptors is maintained, albeit possibly reduced. The lack of stimulatory effect in *ankk1^27ins^* mutants suggests disruption of action of apomorphine at post-synaptic sites. As both D1 and D2 receptors modulate the startle response (Halberstadt and Geyer, 2009), loss of a stimulatory effect of apomorphine on startle response may result from disruption of non-drd2 signaling in *ankk1^27ins^* mutants, possibly as a result of reduced expression of *drd4* or *drd5* as suggested by our qPCR data, or as a secondary effect of loss of drd2 signaling. The further reduction in response to acoustic startle seen in the presence of apomorphine in mutants might reflect action at residual (pre- or post-synaptic) D2 receptors, or again actions of apomorphine at non-D2 receptors.

Further studies employing more specific dopamine receptor agonists and antagonists in conjunction with analysis of drd2 binding affinities and additional immunohistochemical analyses are needed to test these hypotheses.

Despite the noticeable difference in size (i.e., smaller cerebral hemispheres) and distinctions that must be considered when establishing translational comparisons [e.g., zebrafish lack the prefrontal cortex, a region involved in high-order functions commonly disrupted in psychiatric disorders (Mueller and Wullimann, 2015)], the general architecture of the key components of the zebrafish central nervous system is highly conserved with that of humans (see Tropepe and Sive, 2003, for review). Here we exemplify how zebrafish can be exploited as a suitable model to study the development and functioning of the vertebrate nervous system, and to interrogate the functional impact of genetic variation relevant for human disease, including psychiatric disease and substance use disorder.

## Conclusion

To our knowledge, this is the first study aiming at the characterization, at both behavioral and molecular levels, of a loss of function line for *ankk1* and its effect on the development and functioning of the dopaminergic system. Although an *Ankk1* mouse knockout line has been recently characterized by Powell et al. (2021), their research was focused on obesity and we did not find further studies characterizing this mutation or its effects on the dopaminergic system. Our findings strengthen the hypothesis of a functional relationship between *ANKK1* and *DRD2*, suggesting that *ANKK1* might be involved in the maintenance of *DRD2* in the cell membrane, rather than in the specification of dopaminergic neurons or establishment of dopaminergic neuron circuits. However, further studies must be conducted to address this question, such as conditional knockout of the *ankk1* gene at later developmental stages. Despite the limitations listed in our discussions, zebrafish represent a powerful model to investigate behaviors and molecular pathways associated with addiction disorders and psychiatric diseases. As the mutation generated is stable, and easy to genotype, this line paves the way to downstream molecular and cellular studies of functionality.

## Data Availability Statement

The original contributions presented in the study are included in the article/Supplementary Material, further inquiries can be directed to the corresponding author/s.

## Ethics Statement

The animal study was reviewed and approved by the Local Animal Welfare and Ethical Review Board at Queen Mary University of London.

## Author Contributions

AL conducted the experiments, analyzed the data, and wrote the manuscript. JG-G designed the study, generated the line, conducted experiments, and analyzed the data. MK helped to design the experiments. JT-P and WH conducted the experiments and analyzed the data. AM and SH conducted the experiments. CB directed the study, designed the experiments, edited the manuscript, and secured funding. EB-N secured funding. All authors contributed to the article and approved the submitted version.

## Conflict of Interest

The authors declare that the research was conducted in the absence of any commercial or financial relationships that could be construed as a potential conflict of interest.

## Publisher’s Note

All claims expressed in this article are solely those of the authors and do not necessarily represent those of their affiliated organizations, or those of the publisher, the editors and the reviewers. Any product that may be evaluated in this article, or claim that may be made by its manufacturer, is not guaranteed or endorsed by the publisher.

## Abbreviations

III: oculomotor nerve
Cans: commissura ansulata
D: dorsal telencephalic area
Dc: central zone of D
Dd: dorsal zone of D
Dl: lateral zone of D
DIL: diffuse nucleus of the inferior lobe
Dm: medial zone of D
Dp: posterior zone of D
D*V*: descending trigeminal root
ECL: external cellular layer of olfactory bulb including mitral cells
GL: glomerular layer of olfactory bulb
Hd: dorsal hypothalamus
ICL: internal cellular layer of olfactory bulb
LLF: lateral longitudinal fascicle
PGZ: periventricular gray zone of the optic tectum
PPa: parvocellular preoptic nucleus anterior part
TelV: telencephalic ventricle
TeO: optic tectum
Tl: torus longitudinalis
TLa: torus lateralis
TSc: central nucleus of torus semicircularis
TTB: tractus tectobulbaris
Val: lateral division of valvula cerebelli
Vam: medial division of valvula cerebelli
Vas: vascular lacuna of area postrema
Vd: dorsal nucleus of ventral telencephalic area
Vp: posterior nucleus of ventral telencephalic area
Vv: ventral nucleus of ventral telencephalic area.
