# Supplementary material for: Ankk1 Loss of Function Disrupts Dopaminergic Pathways in Zebrafish
Source: Front Neurosci. 2022 Feb 8;16:794653. doi: 10.3389/fnins.2022.794653 (PMC8861280; doi:10.3389/fnins.2022.794653)
Supplement: Supplementary file 1 [file Table_1.pdf]

Supplementary Table 1. List of primers used for No-Go mRNA decay and qPCR analysis.

Gene (purpose)	gene acronym	Accession number	Sequence (5' to 3')	Product size (bp)	Efficiency (%)	Source
actin, β 2 (housekeeping)	*actb2*	NM_181601.5	Forward: CGAGCTGTCTTCCCATCCA<br>Reverse: TCACCAACGTAGCTGTCTTTCTG	86	95	https://doi.org/10.3389/fnbeh.2015.00271
ribosomal protein L13a (housekeeping)	*rpl13a*	NM_212784.1	Forward: TCTGGAGGACTGTAAGAGGTATGC<br>Reverse: AGACGCACAATCTTGAGAGCAG	148	80	https://doi.org/10.3389/fnbeh.2015.00271
eukaryotic translation elongation factor 1 alpha 1, like 1 (housekeeping)	*ef1*	NM_131263.1	Forward: CTGGAGGCCAGCTGAAACAT<br>Reverse: ATCAAGAAGAGTAGTACCGCTAGCATTAC	87	88.4	https://doi.org/10.3389/fnbeh.2015.00271
ankyrin repeat and kinase domain containing 1	*ankk1*	NM_001130665.1	Forward: CCTGAGTGCGAGGACATGAT<br>Reverse: CTGTTCAGGCAGACGCTTTC	168	91.85	own produced
dopamine receptor receptor 1 (dopaminergic system)	*drd1*	NM_001135976.2	Forward: TGGTTCCTTTCTGCAACCCA<br>Reverse: AGTGATGAGTTCGCCCAACC	100	84.3	https://doi.org/10.7554/eLife.51295
dopamine receptor receptor 2a (dopaminergic system)	*drd2a*	XM_009291617.3 and XM_005157501.4	Forward: TCCACAAAATCAGGAAAAGCGT<br>Reverse: CAGCCAATGTAAACCGGCAA	106	100.17	https://doi.org/10.7554/eLife.51295

dopamine receptor receptor 2b (dopaminergic system)	*drd2b*	NM_197936.1	Forward: GCACGGCCAGCATTCTTAATC<br>Reverse: GAAAGCACCCAAACAACGGA	136	98.6	own produced
dopamine receptor receptor 3 (dopaminergic system)	*drd3*	XM_021470111.1 and XM_005162673.4	Forward: ATCGAGTTTCGCAGAGCCTT<br>Reverse: TCCACAGTGTCTGAAAGCCG	95	91.09	https://doi.org/10.7554/eLife.51295
dopamine receptor receptor 4a (dopaminergic system)	*drd4a*	NM_001012616.3	Forward: GCCTCTTTCCCATCTCACAG<br>Reverse: CTCAGACACACCAGCACGTT	281	93.56	https://doi.org/10.1016/j.cbpc.2017.01.010
dopamine receptor receptor 4b (dopaminergic system)	*drd4b*	NM_001012618.1	Forward: AGACCCCAGTGAATGCAA<br>Reverse: AGTTTGGCTTTGCGTGTT	150	109.8	https://doi.org/10.1016/j.cbpc.2017.01.010
dopamine receptor receptor 5 (dopaminergic system)	*drd5*	XM_003199767.5	Forward: TCCAGCACAAACGCACAGAGAG<br>Reverse: TCC AGC ACA CAC ACA CAC ACA C	156	89.51	own produced
dopamine transporter (dopaminergic system)	*slc6a3*	NM_131755.1	Forward: GCCTGGTTTTACGGAGTGGA<br>Reverse: GGAGGATTGAAGGTGGCGAA	152	118.34	https://doi.org/10.7554/eLife.51295
dopamine β hydroxylase (dopaminergic system)	*dbh*	NM_001109694.2	Forward: GAGGAGGTCAAGAGGTGGAGG<br>Reverse: CATCGCCTGGCAGAACAGTCAC	105	104.54	own produced
