# Supplementary material for: Ankk1 Loss of Function Disrupts Dopaminergic Pathways in Zebrafish
Source: Front Neurosci. 2022 Feb 8;16:794653. doi: 10.3389/fnins.2022.794653 (PMC8861280; doi:10.3389/fnins.2022.794653)
Supplement: Supplementary file 2 [file Table_2.pdf]

Supplementary Table 2. Statistics (log 10) for gene expression data.

	ankk1$^{+/+}$		***ankk1***$^{+/27ins}$		***ankk1***$^{27ins/27ins}$			
**Gene name**	**Mean**	**SD**	**Mean**	**SD**	**Mean**	**SD**	**F (DFn, DFd)**	***p***
ankk1	3.99351E-07	0.5807945	-4.91904	1.610936	-6.925562	3.41016	F (2, 11) = 13,86	**0.001***
drd1	-6.55651E-08	5.141269	6.199594	2.550406	-0.3409315	0.8468519	F (2, 11) = 5,336	0.024
drd2a	-4.05312E-07	2.04459	0.5226158	1.439238	-0.9210827	1.731454	F (2, 11) = 0,7566	0.4922
drd2b	1.18613E-06	0.8386233	-0.2607388	1.32328	5.354655	0.6617051	F (2, 11) = 42,56	**< 0,0001***
drd3	-1.39475E-06	0.7111623	0.3791922	1.215033	-3.205326	6.092118	F (2, 11) = 1,535	0.2582
drd4a	1.43051E-06	4.102798	1.682233	2.357038	-0.5451202	0.7565644	F (2, 11) = 0,7572	0.4919
drd4b	4.52995E-07	2.643887	-1.410188	2.102053	-5.62743	5.208304	F (2, 11) = 3,213	0.0796
drd5	5.48363E-07	4.678932	-2.098101	3.161005	-4.780843	2.992601	F (2, 11) = 1,809	0.2093
slc6a3	-3.57628E-07	3.790946	1.426936	2.386752	-0.6109747	1.874648	F (2, 11) = 0,6118	0.5598
dbh	-4.05312E-07	1.873834	0.903142	1.067044	0.6820229	2.032615	F (2, 11) = 0,3888	0.6868

Legend: * significant effects.
