# Supplementary material for: Ankk1 Loss of Function Disrupts Dopaminergic Pathways in Zebrafish
Source: Front Neurosci. 2022 Feb 8;16:794653. doi: 10.3389/fnins.2022.794653 (PMC8861280; doi:10.3389/fnins.2022.794653)
Supplement: Supplementary file 3 [file Table_3.pdf]

Supplementary Table 3. P value adjustment for qPCR on 5dpf zebrafish larvae: Tukey method for multiple comparisons within groups with *emmeans* R package (R Core Team, 2017).

**Gene:** ***ankk1 (larvae)***					
**Contrast**	**Estimate**	**SE**	**Df**	**t.ratio**	**p.value**
WT - HOM	6.926	1.85	110	3.737	0.0009
WT - HET	4.919	1.75	110	2.816	0.0158
HOM -HET	-2.007	1.85	110	-1.083	0.5267
**Gene:** ***dat (larvae)***					
**Contrast**	**Estimate**	**SE**	**Df**	**t.ratio**	**p.value**
WT - HOM	0.611	1.85	110	0.330	0.9417
WT - HET	-1.427	1.75	110	-0.817	0.6935
HOM -HET	-2.038	1.85	110	-1.100	0.5163
**Gene:** ***dbh (larvae)***					
**Contrast**	**Estimate**	**SE**	**Df**	**t.ratio**	**p.value**
WT - HOM	-0.682	1.85	110	-0.368	0.9281
WT - HET	-0.903	1.75	110	-0.517	0.8633
HOM -HET	-0.221	1.85	110	-0.119	0.9922
**Gene:** ***drd1 (larvae)***					
**Contrast**	**Estimate**	**SE**	**Df**	**t.ratio**	**p.value**
WT - HOM	0.341	1.85	110	0.184	0.9815
WT - HET	-6.200	1.75	110	-3.548	0.0016
HOM -HET	-6.541	1.85	110	-3.530	0.0018
**Gene:** ***drd2a (larvae)***					
**Contrast**	**Estimate**	**SE**	**Df**	**t.ratio**	**p.value**
WT - HOM	0.921	1.85	110	0.497	0.8729
WT - HET	-0.523	1.75	110	-0.299	0.9519
HOM -HET	-1.444	1.85	110	-0.779	0.7166
**Gene:** ***drd2b (larvae)***					
**Contrast**	**Estimate**	**SE**	**Df**	**t.ratio**	**p.value**
WT - HOM	-5.355	1.85	110	-2.890	0.0128
WT - HET	0.216	1.75	110	0.149	0.9878
HOM -HET	5.615	1.85	110	3.030	0.0085
**Gene:** ***drd3 (larvae)***					
**Contrast**	**Estimate**	**SE**	**Df**	**t.ratio**	**p.value**
WT - HOM	3.205	1.85	110	1.730	0.1988
WT - HET	-0.379	1.75	110	-0.217	0.9744
HOM -HET	-3.585	1.85	110	-1.934	0.1339
**Gene:** ***drd4a (larvae)***					
**Contrast**	**Estimate**	**SE**	**Df**	**t.ratio**	**p.value**
WT - HOM	0.545	1.85	110	0.294	0.9534
WT - HET	-1.682	1.75	110	-0.963	0.6018
HOM -HET	-2.227	1.85	110	-1.202	0.4546
**Gene:** ***drd4b (larvae)***					
**Contrast**	**Estimate**	**SE**	**Df**	**t.ratio**	**p.value**
WT - HOM	5.627	1.85	110	3.037	0.0083
WT - HET	1.410	1.75	110	0.807	0.6994
HOM -HET	-4.217	1.85	110	-2.276	0.0635
**Gene:** ***drd5 (larvae)***					
**Contrast**	**Estimate**	**SE**	**Df**	**t.ratio**	**p.value**
WT - HOM	4.781	1.85	110	2.580	0.0299
WT - HET	2.098	1.75	110	1.201	0.4552
HOM -HET	-2.683	1.85	110	-1.448	0.3202
**Gene:** ***drd2a (adult)***					
**Contrast**	**Estimate**	**SE**	**Df**	**t.ratio**	**p.value**
WT - HOM	-1.928	1.88	30	-1.027	0.5657

WT - HET	-1.141	1.88	30	-0.608	0.8170
HOM -HET	0.788	1.88	30	0.420	0.9078
**Gene: *drd2b (adult)***					
**Contrast**	**Estimate**	**SE**	**Df**	**t.ratio**	**p.value**
WT - HOM	-8.232	1.88	30	-4.386	0.0004
WT - HET	-4.127	1.88	30	-2.199	0.0877
HOM -HET	4.104	1.88	30	2.187	0.0899
