# Supplementary material for: Ankk1 Loss of Function Disrupts Dopaminergic Pathways in Zebrafish
Source: Front Neurosci. 2022 Feb 8;16:794653. doi: 10.3389/fnins.2022.794653 (PMC8861280; doi:10.3389/fnins.2022.794653)
Supplement: Supplementary file 4 [file Image_1.pdf]

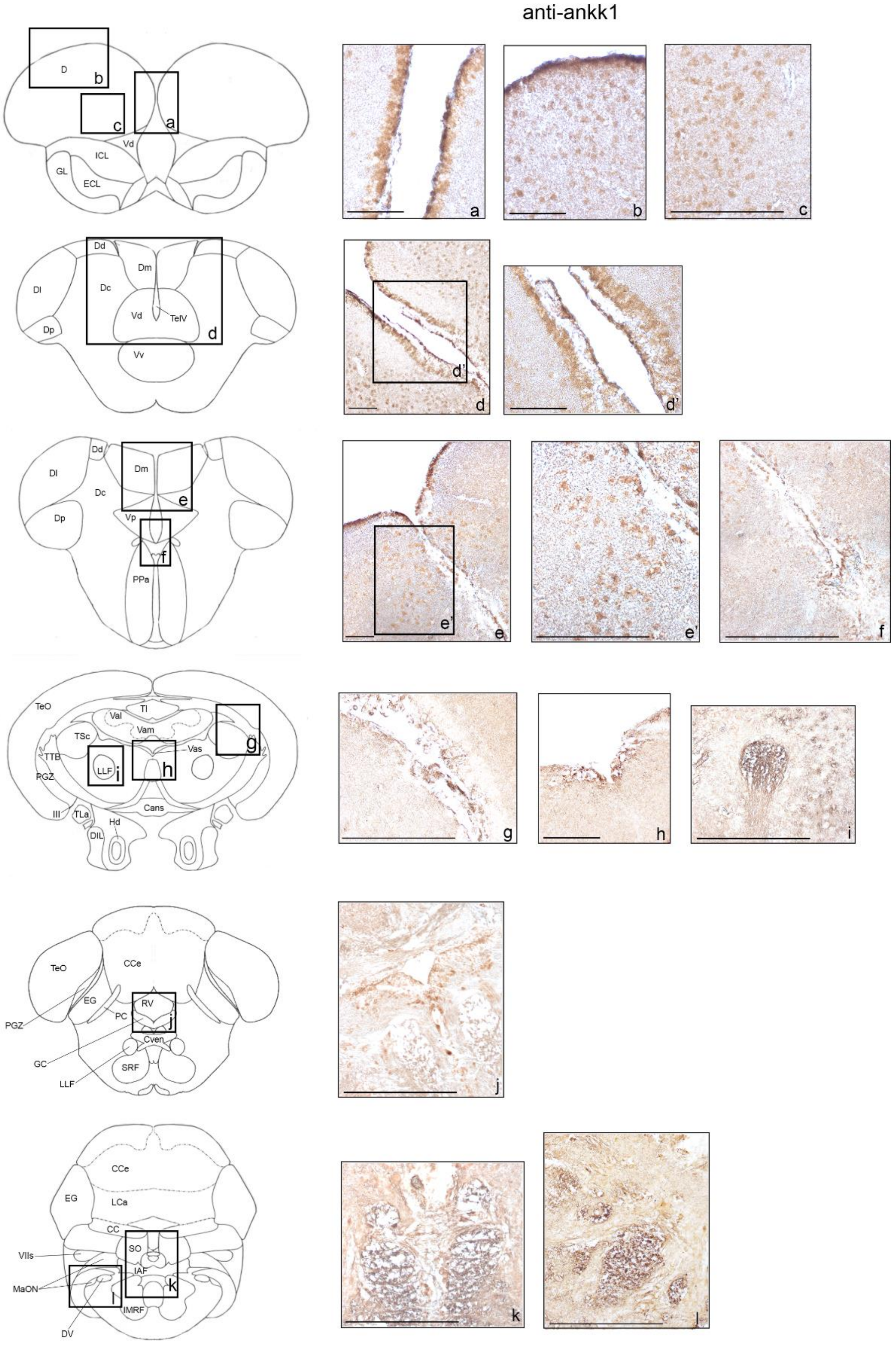

Supplementary Figure 1. Ankk1 immunohistochemistry in adult wild type zebrafish brain. On the right, ankk1 protein distribution in transverse brain sections. On the left, schematic depiction of zebrafish brain, transversal section (adapted from Wullimann et al.,

1996). (**a-f**) Positive staining in the forebrain; (**g-i**) positive staining in the midbrain; (**j-l**) positive staining in the hindbrain. Scale bars: d, e, 50 µm; a, b, d', h, 100 µm; c, e', f, g, i, j, k, l, 200 µm. Abbreviations: III, oculomotor nerve; Cans, commissura ansulata; D, dorsal telencephalic area; Dc, central zone of D; Dd, dorsal zone of D; DIL, diffuse nucleus of the inferior lobe; Dl, lateral zone of D; Dm, medial zone of D; Dp, posterior zone of D; D*V*, descending trigeminal root; ECL, external cellular layer of olfactory bulb including mitral cells; GC, central griseum; GL, glomerular layer of olfactory bulb; Hd, dorsal hypothalamus; IAF, inner arcuate fibers; IMRF, intermediate reticular formation; ICL, internal cellular layer of olfactory bulb; LLF, lateral longitudinal fascicle; MaON, magnocellular octaval nucleus; PGZ, periventricular gray zone of the optic tectum; PPa, parvocellular preoptic nucleus, anterior part; SO, secondary octaval population; TelV, telencephalic ventricle; TeO, optic tectum; Tl, torus longitudinalis; TLa, torus lateralis; TSc, central nucleus of torus semicircularis; TTB, tractus tectobulbaris; Val, lateral division of valvula cerebelli; Vam, medial division of valvula cerebelli; Vas, vascular lacuna of area postrema; Vd, dorsal nucleus of ventral telencephalic area; Vp, posterior nucleus of ventral telencephalic area; Vv, ventral nucleus of ventral telencephalic area; *VII*s, sensory root of the facial nerve.
