# Supplementary material for: Ankk1 Loss of Function Disrupts Dopaminergic Pathways in Zebrafish
Source: Front Neurosci. 2022 Feb 8;16:794653. doi: 10.3389/fnins.2022.794653 (PMC8861280; doi:10.3389/fnins.2022.794653)
Supplement: Supplementary file 6 [file Image_3.pdf]

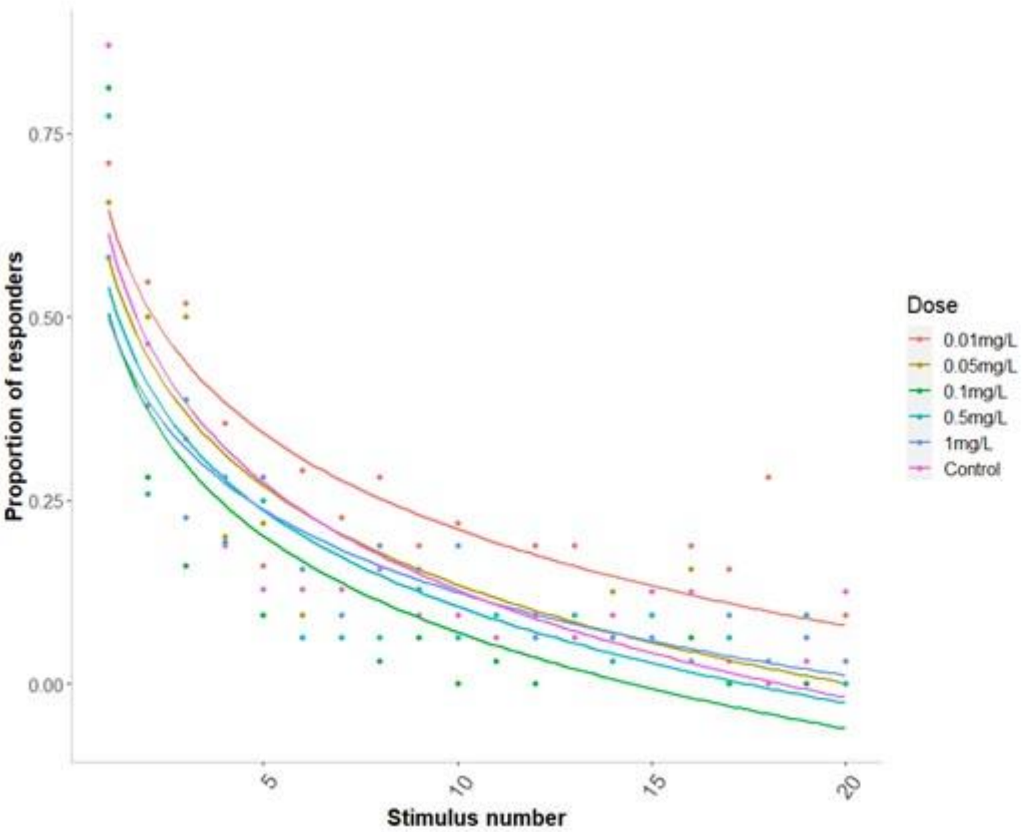

Supplementary Figure 3. Habituation response of wild type fish in the presence and absence of amisulpride. Dose response curve showing the habituation rate to repeated acoustic stimuli (startles) on 5 days post fertilization wild type fish using six different doses of amisulpride. N = 32 per group.
