# Supplementary figures and images for: Ankk1 Loss of Function Disrupts Dopaminergic Pathways in Zebrafish
Source: Front Neurosci. 2022 Feb 8;16:794653. doi: 10.3389/fnins.2022.794653 (PMC8861280; doi:10.3389/fnins.2022.794653)

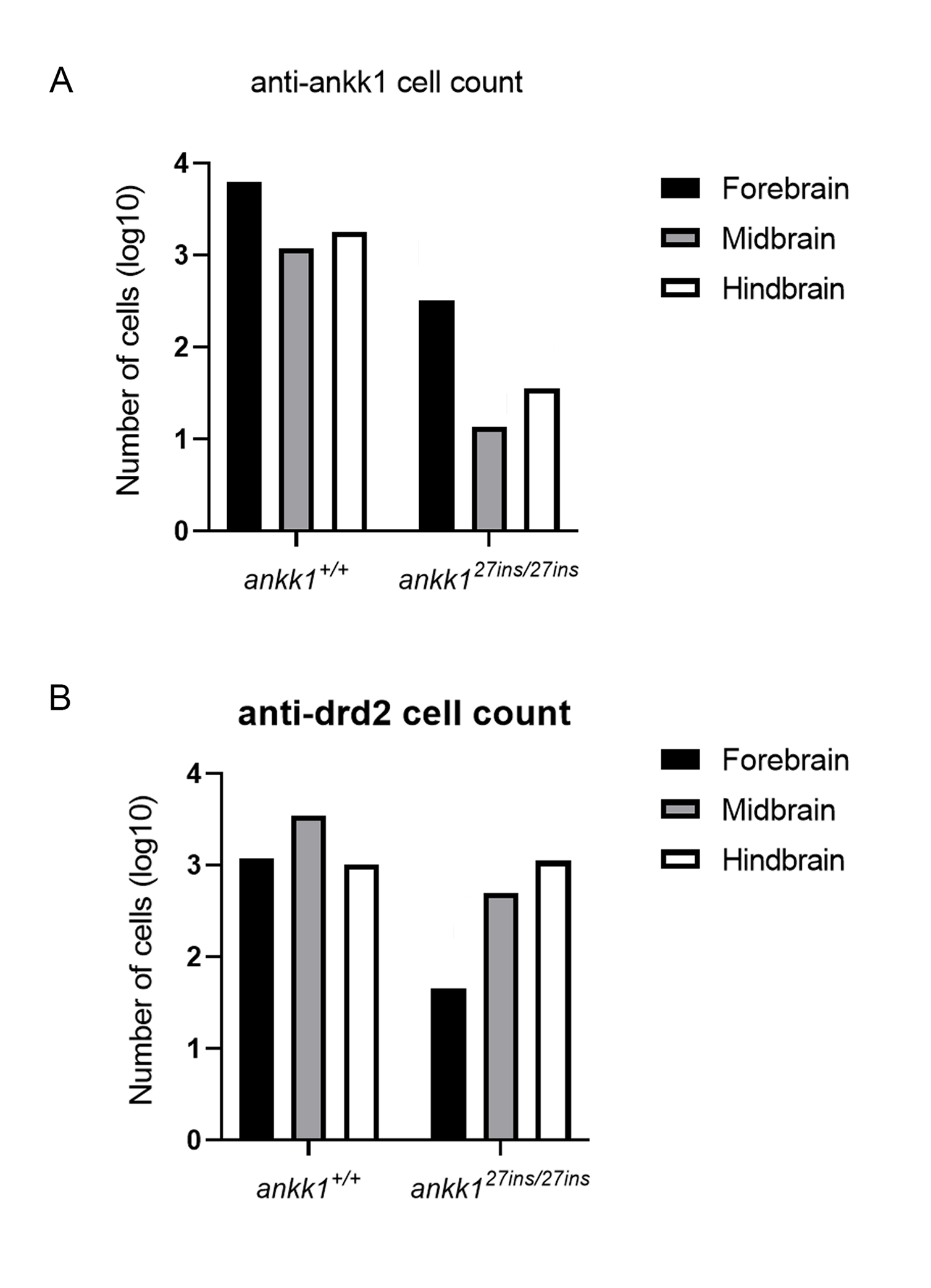

Supplement: Supplementary file 7 [file Image_4.TIF]
